# Immobilization and Controlled-Release Studies of Bovine Serum Albumin Using Empty Fruit Bunch Date Palm-Based Hydrogel Synthesized via Microwave Radiation

**DOI:** 10.3390/polym18070852

**Published:** 2026-03-31

**Authors:** Yousef M. Alanazi, Abdullah Al Ragib, Mohamed Aboughaly, Chun-Yang Yin, Mohanad El-Harbawi

**Affiliations:** 1 Department of Chemical Engineering, King Saud University, Riyadh 11421, Saudi Arabia; yalanazi1@ksu.edu.sa (Y.M.A.); ragibnstu@gmail.com (A.A.R.); 2 MMRI, Department of Chemical Engineering, McMaster University, Hamilton, ON L8S 4L7, Canada; abougm1@mcmaster.ca; 3 Center for Materials Innovation and Technology and College of Engineering and Computer Sciences, VinUniversity, Hanoi 100000, Vietnam; chunyang.y@vinuni.edu.vn

**Keywords:** bovine serum albumin, immobilization, controlled release, empty fruit bunch date palm, hydrogel, microwave

## Abstract

The synthesis of sustainable and promising biomaterials for biomedical applications has recently gained increasing importance. In this study, a hybrid hydrogel was synthesized from empty palm date bunches through the blending of natural (carboxymethyl cellulose) and synthetic polymers (polyvinyl alcohol, polyvinylpyrrolidone) using both traditional and microwave-assisted methods. The aim was to investigate the ability of the hydrogel to immobilize and control the release of bovine serum albumin (BSA), a model protein widely used in pharmaceutical biotechnology. The effect of key parameters such as pH, temperature and hydrogel dosage on protein immobilization was investigated. Optimal results were observed at a pH of 7.4, a temperature of 37 °C and a dosage of 2 g/L—such conditions are very close to the human physiological environment. Kinetic and isotherm models indicated that the immobilization process adhered to pseudo-second-order kinetics and was well-fitted to the Langmuir isotherm. This implied a monolayer adsorption mechanism on a comparatively homogeneous surface. The release studies demonstrated a time-dependent and diffusion-controlled trend, with BSA attaining equilibrium release at 150 min. Overall, the results underline the potential of the microwave-synthesized plant-based hydrogel as a promising material for controlled drug delivery and other biomedical applications due to its efficiency and sustainability.

## 1. Introduction

In recent years, hydrogels have garnered substantial attention as useful biomaterials due to their three-dimensional framework, excellent water-retention capacity and customizable physicochemical properties. To address the limitations of single-component hydrogels, hybrid systems integrating natural and synthetic polymers are extensively studied for enhanced mechanical strength, biocompatibility and stability. Carboxymethyl cellulose (CMC), a biodegradable and hydrophilic polysaccharide, possesses exceptional biocompatibility while frequently requiring reinforcement to increase its structural integrity. Polyvinyl alcohol (PVA) and polyvinyl pyrrolidone (PVP) are synthetic polymers often integrated with natural matrices to provide elasticity, chemical resistance and mechanical sturdiness. The use of crosslinking agents such as citric acid (CA) and glutaraldehyde (GA) further stabilizes the polymeric network by introducing covalent bonds that control swelling, porosity and durability properties.

Conventionally, hybrid hydrogel synthesis is produced via conventional hotplate heating which promotes homogeneous mixing and crosslinking of polymer blends. Recently, microwave-assisted heating has emerged as a quick and energy-efficient alternative that offers increased reaction kinetics and uniform heating [1,2]. For instance, Fadl et al. [3] reported on microwave-induced grafting to prepare hydrogels that have superior properties and were efficient for dye removal. Verma and Pradhan [4] developed a biodegradable, biocompatible hydrogel from starch and hydroxypropylmethylcellulose with citric acid as natural cross-linker by microwave irradiation. The hydrogel exhibited excellent swelling properties, prolonged stability and reduced hemolytic activity. Microwave irradiation affords quick and volumetric heating which facilitates more uniform crosslinking and network formation compared to conventional conductive heating, which is frequently inhibited by slower heat transfer and temperature gradients. By using both methods, the comparative effect of processing technique and crosslinking chemistry on the structural, mechanical and functional properties of CMC–PVA–PVP hydrogels can be studied for potential applications in biomedical, pharmaceutical and environmental fields. For example, CMC/PVA hydrogel films were synthesized for the prolonged delivery of water-soluble basic drugs using citric acid as an inexpensive and non-toxic crosslinking agent [5]. Likewise, the effectiveness of PVA/CMC/attapulgite clay hydrogels for wound dressing was shown in a recent study [6], where CA was used as the crosslinking agent to establish hemolysis, protein adsorption and antimicrobial activity. In another development, Kumar and Paik [7] investigated the protein immobilization and stability properties of heterogeneous hollow mesoporous nanocapsules (Hhmn) for drug and protein delivery, in which they discovered that BSA adsorption capacity increased with the increase in the initial concentration of BSA.

In our previously reported study [8], we developed a hybrid hydrogel through the blending of natural (CMC) and synthetic polymers (PVA, PVP) in the presence of two varieties of crosslinking agents (CA/GA) via the conventional hotplate method combined with a microwave-assisted heating technique. In our current study, we investigated the application of our novel hydrogel in the field of pharmaceutical biotechnology by studying the immobilization and controlled release of bovine serum albumin (BSA). BSA is a globular protein produced from cow’s blood serum and extensively used in biochemical, pharmaceutical and biomedical research as a standard, stabilizer and carrier molecule. To the best of our knowledge, our work represents one of the first efforts to employ microwave-assisted technology for the preparation of a hydrogel derived from date palm biomass and subsequently to utilize this novel material for the immobilization and controlled release of BSA. Many current BSA immobilization systems use non-sustainable synthetic materials. This study addresses this by developing a microwave-assisted, EFB-derived hydrogel that offers customizable immobilization–release performance within a sustainable biomass-based platform. While previous studies have researched hydrogel synthesis from numerous natural and synthetic polymers, none have specifically addressed the valorization of empty fruit bunches from date palm (agricultural byproduct) through a sustainable microwave-assisted method that not only increases efficiency but also reduces environmental impact. By incorporating microwave irradiation with biomass-derived CMC and blending it with synthetic polymers such as PVA and PVP, our study introduces a unique hybrid hydrogel with favorable physicochemical properties compared to those synthesized using traditional methods. In addition, applying hydrogel with data palm biomass-based components for BSA immobilization and release introduces a novel dimension to biomedical research which highlights its potential applications in drug delivery systems and therapeutic protein delivery.

## 2. Methodology

### 2.1. Immobilization Equilibrium Study

The hydrogel was synthesized as per our previous study [8]. Briefly, CMC was blended with synthetic polymers (PVA and PVP), using CA (20% *w*/*v*) as a crosslinking agent. High-frequency microwave technology (TANK eco, Sineo, Shanghai, China) was used to accelerate the process and prepare a hydrogel polymer network in a short time. A mixture of CMC, PVA and PVP was prepared to create polymer blends by maintaining different ratios of CMC to (PVA + PVP) (10/90, 20/80, 30/70, 40/60, 50/50). First, a viscous solution of CMC (2% *w*/*v*) was prepared by dissolving 1 g of CMC powder in 50 mL of deionized water under strong agitation at 60 °C until fully dissolved (approximately 1 h). Next, a PVA solution (10% *w*/*v*) was prepared by adding 5 g of PVA powder to deionized water at 80 °C for 1 h and a PVP solution (10% *w*/*v*) was prepared by adding 5 g of PVP powder to 50 mL of deionized water with manual stirring at room temperature. Subsequently, CA (20% *w*/*v*) was added dropwise to crosslink CMC, PVA and PVP. Then the mixture was poured in a 100 mL Teflon vessel and the vessel was subjected to microwave irradiation at 500 W and 120 °C for varying durations (3–7 min). After microwave heating, the viscous solutions were slowly poured into petri dish molds (90 mm diameter) and left to dry overnight at 37 °C in an oven dryer. Distilled water was used to neutralize the acidic dry hydrogel films. After drying, the hydrogel film was stored in a desiccator for further use.

The immobilization equilibrium study was conducted via a batch technique as a function of time (up to 6.5 h) by immersing the hydrogel samples in 50 mL of the 100 ppm (initial concentration) BSA-buffer solutions at 37 °C. For the immobilization technique, phosphate buffer solution (pH 7.4) was used. Subsequently, 100 mg of dried hydrogel pieces were added to glass Erlenmeyer flasks containing 50 mL phosphate buffer solution for the immobilization process for ten different hydrogels. The initial and final concentration measurements of BSA protein as well as concentration at each time interval were calculated by recording absorbance in UV-Vis spectrophotometer at 280 nm wavelength and data from the standard calibration curve. To negate potential interference from hydrogel leachates or residual polymers during UV–Vis quantification at 280 nm, blank hydrogel samples (without BSA) were incubated under identical experimental conditions and analyzed as controls. The absorbance of these blanks was negligible and subtracted where necessary to ensure that no significant spectral interference affected BSA determination. After measuring the optical density (absorbance) of BSA protein solution at the same wavelength, the amount of immobilized BSA was determined using UV-vis spectrometer and calculated using the following equation:

(1) $$q_{e}=\;\frac{\left(C_{0}-C_{e\;}\right)V}{m}$$

where q_e_ is the BSA immobilization capacity, or the final equilibrium of BSA protein immobilization by the hydrogel (mg/g); C_o_ and C_e_ are the initial and equilibrium BSA concentrations (mg/L); V is the solution volume of the buffered BSA used (L); and m is the mass of the dried hydrogel (g). Immobilization capacity establishes the capacity of hydrogel to immobilize BSA per gram of surface area.

The immobilization capacities of ten different hydrogels were analyzed for BSA immobilization using a batch process. To evaluate the effects of pH on immobilization capacity of hydrogels, the pH of the point zero charge of the hydrogel (pH_pzc_) was investigated. In each beaker, 30 mL of a 0.05 M NaCl solution was added. The pH of the NaCl solutions was subsequently adjusted to 6, 7, 8 and 9 by adding either 0.1 M HCl or 0.1 M NaOH. The solutions were added to Erlenmeyer flasks once the pH had been set and the initial pH (pH_i_) had been measured. Then, 50 mg of each hydrogel was immersed in the solutions and shaken manually and maintained for 24 h. The final pH (pH_f_) of the supernatant solution was recorded. The ΔpH (pH_i_-pH_f_) measurement in relation to pH_i_ plot was used to establish the points of zero charge. To examine the effects of pH in BSA immobilization, 10 mg of BSA powder was mixed with 100 mL of buffer solution to prepare 100 ppm of BSA-buffer solution. A total of 10 beakers with each containing 10 mL BSA-buffer solution were prepared, where 10 different formulations of hydrogel were subjected to submerge for 150 min at 37 °C. Each hydrogel weighed 20 mg to maintain the dosage at 2 g/L. The immobilization capacity was calculated from the recorded absorbance data at three different pH levels (6.4, 7.4 and 8.4).

This experiment was conducted under the following conditions: temperature 37 °C and pH 7.4. The initial concentration was adjusted from 50 ppm to 300 ppm. Different amounts of BSA (5 to 30 mg) were mixed separately with 100 mL of buffer solution to prepare 50, 100, 150, 200, 250, and 300 ppm of BSA-buffer solution. A total of 10 beakers, each containing 10 mL of a 50 ppm BSA-buffer solution, were maintained, and 10 different formulations of hydrogels were submerged for 150 min. The same procedure was applied up to an initial concentration of 300 ppm. The immobilization capacity of the hydrogels was recorded and further plotted against six different initial concentrations of BSA-buffer solution.

The immobilization capacities of ten different hydrogels were investigated under the three different temperatures of 24, 37 and 42 °C. A total of 10 mg of BSA powder was mixed with 100 mL of buffer to prepare 100 ppm of BSA-buffer solution, and 10 mL of this solution was maintained in each of the 10 beakers to react with 20 mg of each hydrogel sample for a period of 150 min. Thus, the dosage was maintained at 2 g/L with pH 7.4. The immobilization capacity of hydrogels was recorded from the absorbance data in UV-Vis spectrometer and plotted against the temperature.

To investigate the effects of dosage of hydrogels on immobilization capacity at a specific temperature of 37 °C and a pH of 7.4, 100 ppm of BSA-buffer solution was prepared by mixing 100 mL of buffer with 10 mg of BSA powder. For each hydrogel sample, three different mass values of hydrogels were used (10 mg, 20 mg and 30 mg). They were immersed into 10 mL of separately kept BSA-buffer solution to assess the immobilization capacity at 1, 2 and 3 g/L dosages for a duration of 150 min. The immobilization capacity of hydrogels was plotted against three different dosages to assess the effects of dosage on BSA immobilization of each hydrogel. To quantify BSA protein concentration in hydrogel immobilization and release studies, a standard BSA calibration curve was established using a UV-Visible/NIR spectrophotometer (Jasco-V-770, Tokyo, Japan) at 280 nm.

### 2.2. Immobilization Kinetics Study

The BSA immobilization capacity at equilibrium was further analyzed to determine the kinetics and feasibility of the immobilization process. Since immobilization occurs through adsorption, the experimental data were evaluated using pseudo-first-order (PFO), pseudo-second-order (PSO) and intra-particular diffusion (IPD) kinetic models. For the kinetics study, the immobilization capacity of BSA at each time interval (q_t_) was subtracted from the equilibrium immobilization capacity (q_e_). With the equilibrium time study and data obtained from the kinetics observation, the immobilization model was constructed using different initial concentrations of BSA buffer solution. When the immobilization rate is influenced by the number of unoccupied binding sites, the process adheres to PFO kinetics. The PFO model can be described by the Lagergren equation:

(2) $$\frac{dq_{t}}{dt}=K_{1}(q_{1e}-q_{t})$$

where $q_{1e}$ and $q_{t}$ refers to immobilization capacity of hydrogel at equilibrium and any time (mg/g), respectively, and $K_{1}$ is the pseudo-first-order rate constant (1/min). Integrating the previous equation for the boundary conditions t = 0 to t = t and $q_{t}=0$ and $q_{t}=q_{t}$ results in the following non-linear equations:

(3) $$q_{t}=q_{1e}(1-e^{-K_{1}t})$$

The non-linear form can be written in a linear form (logarithmic), and the transformation produces the equation below:

(4) $$\log(q_{1e}-q_{t})=log(q_{1e})-\frac{K_{1}}{2.303}t$$

When the bonding between the hydrogel surface and BSA happens via covalent or ionic interactions, chemisorption becomes the rate-limiting step, consistent with pseudo-second-order kinetics. According to this model, the immobilization rate is proportional to the fraction of accessible binding sites, and it is represented by the following equation:

(5) $$\frac{dq_{t}}{dt}=K_{2}{\left(q_{2e}-q_{t}\right)}^{2}$$

Integrating the previous equation for boundary conditions t = 0 to t = t and $q_{t}=0$ and $q_{t}=q_{t}$ results in the following linear equation:

(6) $$\frac{t}{q_{t}}=\frac{1}{K_{2}{q_{2e}}^{2}}+\frac{1}{q_{2e}}t$$

Equation (6) can be written in a non-linear form:

(7) $$\frac{t}{q_{t}}=\;\frac{1+q_{2e}K_{2}t}{K_{2}{q_{2e}}^{2}}$$

(8) $$q_{t}=\frac{K_{2}{q_{2e}}^{2}t}{1+q_{2e}K_{2}t}$$

The IPD model, also called the Weber–Morris model, is frequently used to describe adsorption kinetics when the rate-limiting step is diffusion of the BSA within the pores of the hydrogel; in this case, the immobilization capacity of hydrogel can be expressed by the following equation:

(9) $$q_{t}=K_{i}\sqrt{t}+\;C$$

where $q_{t}$ is the amount of BSA immobilized at time (mg/g), $\;K_{i}$ is the intra-particle diffusion rate constant ($\frac{mg/g}{{min}^{-1/2}}$) and C is the intercept related to the boundary-layer thickness.

### 2.3. Isotherm Models

Immobilization studies were conducted in batch by separately immersing the hydrogel samples in 50 mL of different concentrations (from 50 to 300 ppm) of BSA in buffer solution. The sample-containing solution was maintained at room temperature up to the equilibrium time, similar to the immobilization equilibrium study. The remaining BSA concentration in each hydrogel-soaked BSA-buffer solution of different concentrations at equilibrium was determined from the absorbance obtained by a UV-vis spectrometer. BSA immobilization was determined using Equation (20) and the isotherms were plotted. The isotherm model is referred to as a relationship between the immobilization capacity (q_e_) (amount of immobilized substance (BSA) per gram unit of immobilizer (hydrogel)) and the equilibrium, while keeping the temperature constant. An isotherm can be expressed by the following equation:

(10) $$\frac{x}{m}=\;fC_{e}$$

In this context, x, m and C_e_ represent the immobilized amount at equilibrium, the mass of the immobilizer (hydrogel) and the equilibrium concentration of the immobilized substance (BSA), respectively. The ratio x/m indicates the amount of immobilized substance per unit gram of immobilizer at equilibrium. The fraction of the total solid surface covered was assumed to be Q, and the available surface area for adsorption was 1-Q. Therefore, the rate of immobilization will be as follows:

(11) $$Rate\;of\;immobilization\;=\;K_{i}(1-Q)C_{e}$$

where $K_{i}$ refers to the proportional constant of immobilization and$\;C_{e}$ is the liquid concentration.

(12) $$Rate\;of\;desorption=K_{d}Q$$

where $K_{d}$ denotes the proportional constant of desorption. When equilibrium is established, the rate of immobilization is equal to the rate of desorption and can therefore be written as follows:

$${K_{i}(1-Q)C_{e}\;=\;K}_{d}Q$$

(13) $$K_{i}C_{e}=K_{d}Q+K_{i}QC_{e}$$

By dividing (13) by $K_{d}$, rearranging and taking $\frac{K_{i}}{K_{d}}$ = $K_{L}\;$ with Q denoted as the fractional surface coverage equal to $\frac{X}{b}$, we obtain the following:

$$\frac{X}{b}=\frac{K_{L}C_{e}}{\left(1+K_{L}C_{e}\right)}$$

where X or q_e_ is the amount of immobilized molecule per g of immobilizer, and b or $q_{m}$ is the maximum immobilization. The non-linear equation is expressed by the following:

(14) $$q_{e}=\frac{q_{m}K_{L}C_{e}}{\left(1+K_{L}C_{e}\right)}$$

In linearized form (after dividing by C_e_ and re-arranging), Equation (14) may be written as

(15) $$\frac{C_{e}}{q_{e}}=\frac{1}{K_{L}q_{m}}+\frac{C_{e}}{q_{m}}$$

Equation (14) is known as the Langmuir equation, where $q_{e}$ is the equilibrium immobilized amount on the surface of the immobilizer (mg/g), and $C_{e}$ is the equilibrium concentration (mg/L). $q_{m}$ (mg/g) and $K_{L}$ (L/mg) are the Langmuir constants related to saturation point immobilization capacity and binding affinity, respectively. The essential characteristics of the Langmuir isotherm can be illustrated as a dimensionless constant separation factor or equilibrium parameter, $R_{L}$, which indicates the shape of the isotherm and is expressed by the following equation:

(16) $$R_{L}=\frac{1}{\left(1+K_{L}C_{0}\right)}$$

where $C_{0}$ is the highest initial concentration of the immobilized particle (mg/L) and $K_{L}$ (L/mg) is the Langmuir constant. The value of $R_{L}$ represents the shape of the isotherm: unfavorable ${(R}_{L}>1),\;linear\;\left(R_{L}=1\right)$, favorable $\left(0<R_{L}<1\right)$ or irreversible ($R_{L}=0)$.

The Freundlich isotherm curve is in exponential form which represents an initial surface adsorption process followed by a condensation effect caused by strong solute–solute interaction [9]. According to the theory, when the concentration value is at an intermediate level, immobilization is directly proportional to concentration raised to the power 1/n. Here, n is a variable > 1:

(17) $$\frac{x}{m}\;\propto \;C^{\frac{1}{n}}$$

When K_f_ is used as a proportional constant or immobilization constant, the following non-linear equation can be expressed:

(18) $$\frac{x}{m}=K_{f}C^{\frac{1}{n}}$$

By taking the Logarithm from both sides, equation 18 may be expressed in linear form as follows:

(19) $$\log\frac{x}{m}=\;\log K_{f\;}+\frac{1}{n}\;\log C$$

The linear regression method is the most extensively applied in the calculations of the model parameters owing to its simplicity [10,11]. However, the linearization process changes the independent or dependent variables. This process could introduce propagated errors to the independent or dependent variables and could cause inaccurate estimations of the parameters [12,13]. Also, the linear regression technique requires the transformation of the actual nonlinear form of the model equations to a linear form. The linearization procedure leads to changes in the error structures, violation of the error variance and normality assumptions of the standard least squares [14], and this leads to bias in the fitted model parameters between linear and nonlinear versions of the model equations [15]. In contrast to linear regression, non-linear regression does not involve model transformation; hence, the error structure is unchanged. Non-linear regression generally involves the minimization or maximization of error distribution between experimental and predicted data based on convergence criteria [16]. Therefore, it can provide consistent and accurate estimations for model parameters [12]. It is of great significance to provide the non-linear solving method for the adsorption kinetics and isotherm models. In this study, a comprehensive analysis of immobilization kinetic models (PFO, PSO, IPD) and isotherm models (Langmuir and Freundlich) was performed with the application of non-linear regression in MATLAB (MATLAB R2019a).

### 2.4. Controlled Release Study

Hydrogel-containing immobilized BSA was further immersed in phosphate buffer solution at pH = 7.4 and 37.0 ± 1.0 °C to test the controlled release of BSA. Using UV–Vis spectrophotometry at 280 nm, aliquots of the solutions were taken at specific time intervals (10, 30, 60, 120, 150, 300 and 330 min) to quantify the amount of BSA released from each hydrogel. The immobilization (%) was calculated using the initial concentration (C_0_) and final concentration (C_F_) of BSA. The percentages of immobilization and release were calculated based on Equations (20) and (21), respectively:

(20) $$Immobilization\;(\%)=\frac{C_{0}-C_{F}}{C_{0}}\times 100\;$$

(21) $$Release\;(\%)=\frac{Released\;amount\;of\;BSA}{Immobilized\;amount\;of\;BSA}\times 100$$

In this study, four distinct hydrogel variants were synthesized using both non-microwave and microwave-assisted techniques. For the microwave-assisted method, three hydrogel variants were prepared, each subjected to different reaction times (3, 5 and 7 min) to determine the appropriate hybrid hydrogel. In this study, 3, 5 and 7 min were chosen as the reaction times for irradiation because preliminary tests showed that reaction times below 3 min resulted in hydrogels with low gel strength, indicating weak polymer crosslinking, and these samples degraded much faster. In contrast, irradiation for more than 7 min resulted in excessively stiff gels with visible burnt areas and a loss of well-defined pore structures on microscopy. The 3–7 min range provided optimal stability and allowed for meaningful performance comparisons. Consequently, by modifying the composition and reaction time, a total of ten hydrogel variants were developed from these four primary formulations.

The selection of these ten hydrogel variants was based on extensive experimentation involving five different mixing ratios of the natural polymer CMC and synthetic polymers PVA and PVP. The polymer compositions were varied in ratios of 10/90, 20/80, 30/70, 40/60 and 50/50 to ensure a comprehensive evaluation of hydrogel properties. As a result, a total of fifty hydrogel formulations were initially prepared, from which ten were shortlisted based on their swelling ratio, gel fraction and overall structural integrity (Table 1). Ten different hydrogels—selected on the basis of swelling kinetics: four samples from each method, one from the non-microwave method and three from the microwave-assisted techniques—were identified for further characterization. To serve as control samples, hydrogels composed of 100% CMC and 100% PVA were individually prepared. However, the 100% CMC hydrogel completely dissolved in water within 35 min, while the 100% PVA hydrogel exhibited insufficient swelling capacity; thus, both were unsuitable for further application. Subsequently, ten primarily selected hydrogels were subjected to immobilization and controlled release studies. Based on their immobilization capacities, release efficiencies and kinetic profiles, the best performing hydrogels were investigated.

## 3. Results and Discussion

### 3.1. Immobilization Study

**Figure 1:**
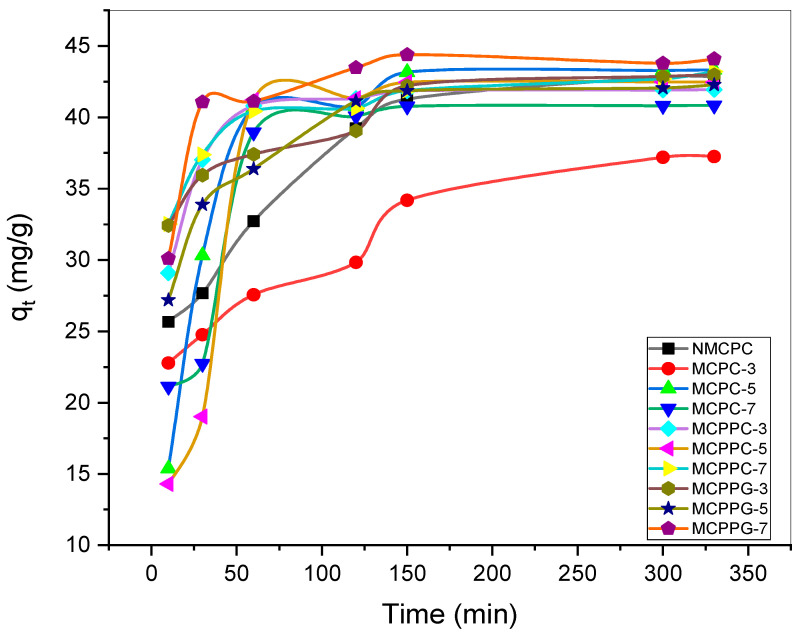
BSA immobilization capacities of the hydrogel samples.

Figure 1 illustrates the BSA immobilization capacities of ten different hydrogels over time while maintaining a BSA concentration of 100 ppm. All hydrogel samples exhibited a rapid increase in immobilization capacity at approximately 150 min, after which the curve gradually plateaued. This trend indicated that BSA molecules rapidly bound to the active sites on the hydrogel surface during the initial phase, followed by a saturation point where no further immobilization occurred, which signified equilibrium. Among the three variations of microwave-assisted hydrogels, MCPC-5, MCPPC-7 and MCPPG demonstrated the highest immobilization capacities, possibly due to their strong cross-linking and the presence of active binding sites within the polymeric network.

**Figure 2:**
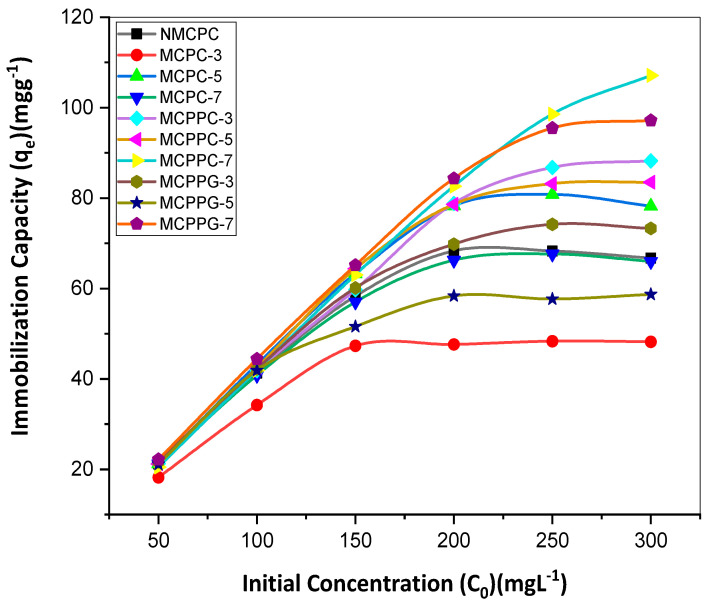
Immobilization capacities at different initial concentrations.

**Figure 3:**
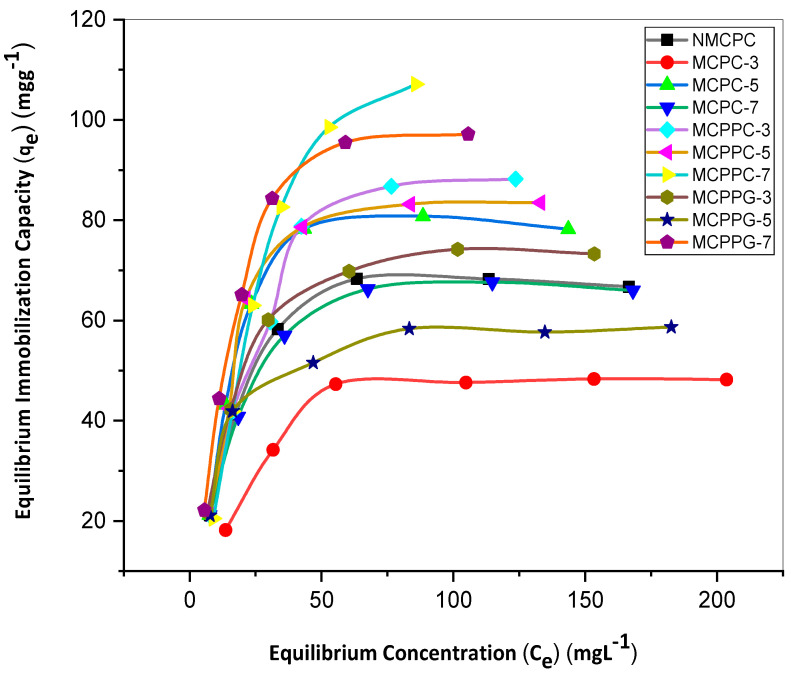
Equilibrium capacity in relation to equilibrium concentration.

Based on the equilibrium-time study, further investigation was conducted to establish the equilibrium capacities of BSA on ten different hydrogel samples at varying BSA concentrations (50 to 300 ppm). This analysis explored the relationship between BSA concentration and the immobilization capacity of hydrogels. As shown in Figure 2, the immobilization capacities increased with rising initial BSA concentration. However, at a certain point, the immobilization capacities became static even when a highly concentrated BSA solution was introduced. Figure 3 demonstrates the relationship between immobilization capacities and concentration at equilibrium in a similar pattern.

**Figure 4:**
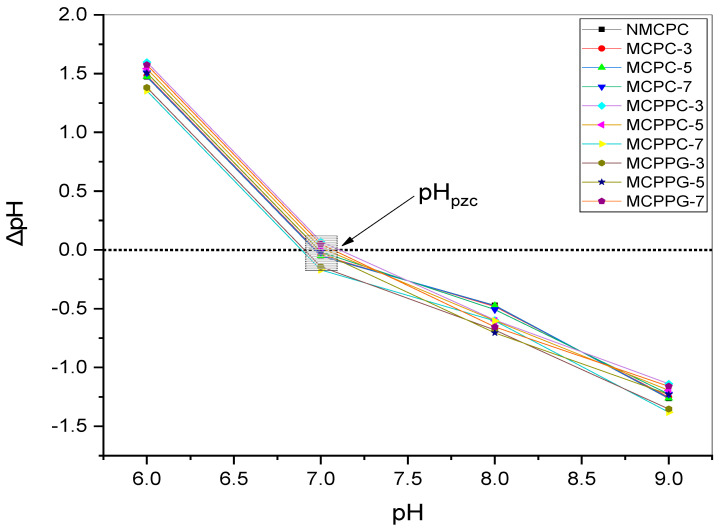
The pH_pzc_ of the hydrogel used for immobilization.

**Figure 5:**
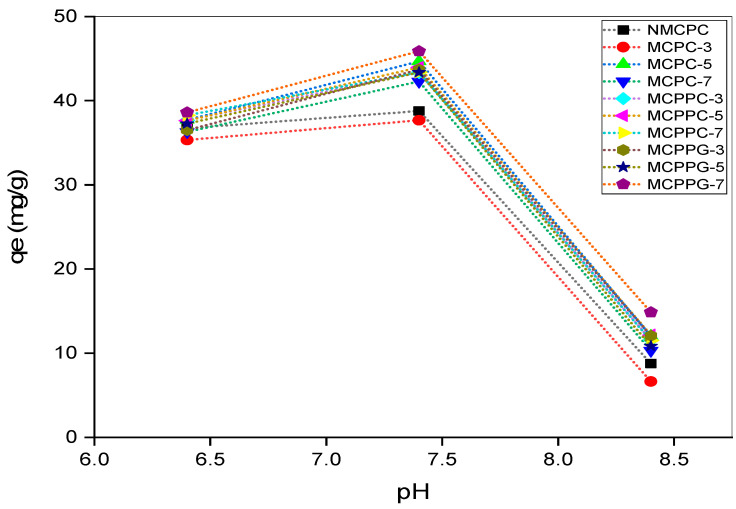
Effect of pH on BSA immobilization.

The isoelectric point (pI) of bovine serum albumin (BSA) ranges between 4.7 and 5.2, indicating that BSA acquires a net negative charge at pH values above this range [17]. In contrast, the hydrogel systems synthesized in this study, i.e., CMC, PVA and PVP, cross-linked with either CA or GA, were found to have a point of zero charge (pH_pzc_) from 6.9 to 7.2 (Figure 4). Therefore, the hydrogel surface is positively charged at pH < 6.9 and becomes negatively charged at pH > 7.2. Electrostatic interactions between the negatively charged BSA and the hydrogel surface plays an important role in protein immobilization. To assess the effect of pH, BSA immobilization experiments were carried out at pH 6.4, 7.4 and 8.4 (Figure 5) with the following conditions: initial concentration 100 ppm, dosage 2 g/L, temperature 37 °C and contact time 150 min. Maximum immobilization capacity was observed at pH 7.4, followed by a moderate level at pH 6.4 and a significant reduction at pH 8.4. At pH 6.4, the hydrogel is positively charged and BSA is negatively charged, which indicates electrostatic attraction. However, BSA at this pH is closer to its isoelectric point and may begin to aggregate or unfold slightly, reducing its solubility and interaction efficiency [17,18]. At pH 7.4, although the hydrogel surface is slightly negative (just above its pH_pzc_), the electrostatic repulsion is weak, and other interactions such as hydrogen bonding, van der Waals forces and hydrophobic interactions may dominate [19]. BSA is also more stable, more soluble and more conformationally favorable for interaction at this near-neutral pH than at pH 6.4 [20]. In addition, hydrogel swelling may be more pronounced at this pH, which enhances the entrapment of BSA molecules [21]. These combined factors likely explain the enhanced immobilization effect at pH 7.4 compared to at pH 6.4, despite the weaker electrostatic attraction. At pH 8.4, both the hydrogel and BSA are strongly negatively charged, rendering significant electrostatic repulsion and markedly reduced immobilization capacities [22,23].

**Figure 6:**
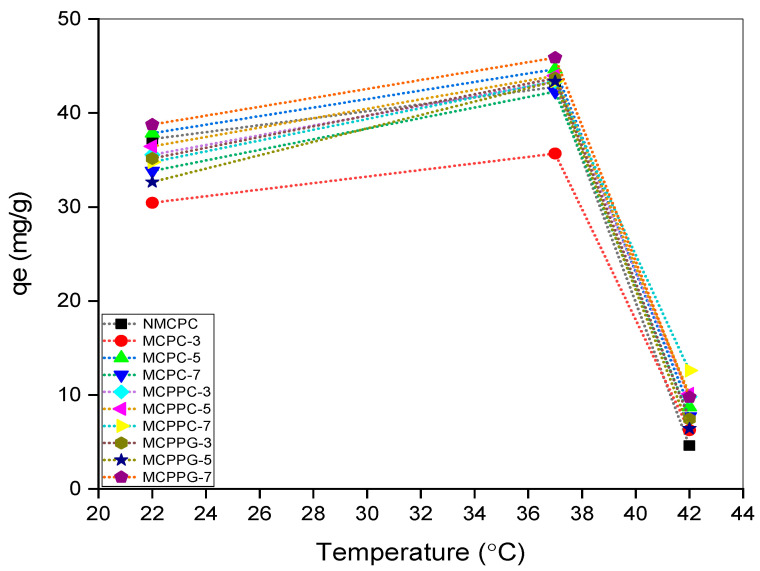
Effect of temperature on BSA immobilization.

The effect of temperature on the immobilization capacities of the hydrogels was assessed at 22, 37 and 42 °C (Figure 6) with the following conditions: initial concentration 100 ppm, dosage 2 g/L, pH 7.4 and contact time 150 min. Among the tested conditions, 37 °C yielded the highest equilibrium adsorption capacity (q_e_) across the majority of the hydrogel samples. This temperature is physiologically relevant and frequently promotes optimal biomolecular interactions, especially protein–hydrogel binding. This was attributed to enhanced molecular mobility without compromising structural integrity [24]. In a different study, increased amounts of BSA were observed on the thermoresponsive polymer-grafted surfaces at 37 °C compared with those at 20 °C because of enhanced hydrophobic interactions with the hydrophobic and thermoresponsive surface [25]. Conversely, at 42 °C, a decrease in qe was observed, possibly due to partial denaturation of BSA or hydrogel softening, which can negatively affect immobilization efficiency. Isolated BSA is reported to partially unfold between 40 and 50 °C which exposes non-polar residues on the surface and facilitates reversible protein–protein interactions [26]. Therefore, the optimal performance at 37 °C suggests that this temperature provides a balance between molecular activity and material stability for effective protein immobilization. Many previous studies have established the best adsorption of BSA at this physiological temperature [27,28].

**Figure 7:**
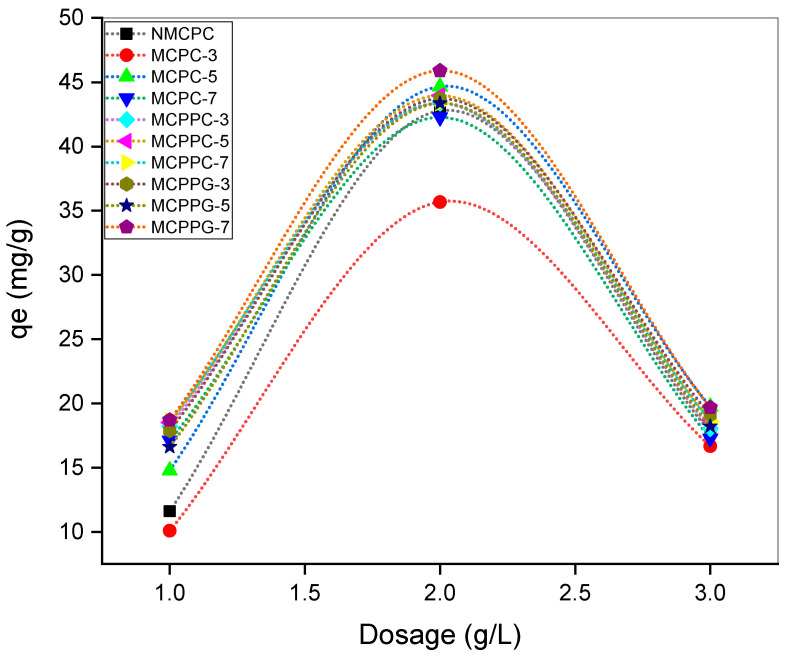
Effect of dosage on BSA immobilization.

To investigate the effect of hydrogel dosage on the immobilization efficiency, varying dosages (1, 2 and 3 g/L) of each hydrogel sample were tested at pH 7.4, temperature 37 °C and BSA concentration of 100 ppm, with a contact time of 150 min. Among the tested dosages, the 2 g/L concentration exhibited the highest immobilization capacity across all hydrogel formulations (Figure 7). This trend suggests that 2 g/L provides an optimal balance between the availability of active binding sites and the steric accessibility of the target molecule. At lower dosages (1 g/L), the reduced amount of hydrogel likely limited the number of available functional groups for effective interaction with BSA, which resulted in a lower immobilization rate. Conversely, at higher dosages (3 g/L), although more binding sites were theoretically available, possible aggregation or overlapping of hydrogel particles might have caused site inaccessibility or diffusion limitations and reduced the immobilization efficiency.

Release experiments were conducted at pH 7.4 to simulate physiological conditions relevant to biomedical applications. The release profile exhibited a measured increase without a marked initial burst effect, which indicated reasonably controlled and sustained protein diffusion from the hydrogel matrix. Even though classical release models were not examined, the time-dependent profile supported diffusion-influenced transport under physiological conditions. Further multi-pH and model-based analyses will be beneficial for extensive mechanistic evaluation.

### 3.2. Immobilization Kinetics

The immobilization capacity at equilibrium time is referred to as the equilibrium capacity (q_e_). Using the data from the equilibrium time and capacity study, further analysis was conducted to establish the mechanism of BSA immobilization on the hydrogel surface. Pseudo-first-order kinetics describes the immobilization rate based on the assumption that it is proportional to the remaining unoccupied binding sites. On the other hand, pseudo-second-order immobilization kinetics is commonly used to model the rate at which BSA immobilizes into or within the hydrogel matrix when the rate-limiting step is assumed to be chemisorption (a strong binding through the valence forces).

The kinetic data for all hydrogel samples were analyzed using the pseudo-first-order model, and the resulting calculated parameters along with statistical indicators are presented in Table 2. Based on statistical evaluation, MCPC-5, MCPPC-3, MCPPC-5 and MCPPG-7 showed the best agreement with the pseudo-first-order model as indicated by high coefficients of determination (R^2^) values, i.e., 0.991, 0.921, 0.913 and 0.951, respectively. There was also a generally low sum of squared errors (SSE) and mean squared error (MSE) values, which confirmed that the predicted data closely adhered to the experimental trend. For these hydrogels, the immobilization process was likely governed by physisorption involving weak interactions between BSA and the hydrogel matrix.

Other samples, including NMCPC, MCPC-3, MCPC-7, MCPPC-7, MCPPG-3 and MCPPG-5, exhibited a weaker correlation with the model with R^2^ values ranging from 0.543 to 0.804. This was coupled with noticeably higher SSE and MSE values, which suggested greater deviation between predicted and actual kinetic behavior. Notably, the Chi^2^ values did not always follow the same trend as R^2^. For example, MCPPC-5 displayed a relatively high R^2^ (0.913) and a higher Chi^2^ value (3.389) suggesting that while the overall trend was well captured, some data points deviated more strongly from the model prediction. In contrast, MCPPC-7 had a lower R^2^ (0.772) but a smaller Chi^2^ (0.471), suggesting that deviations were small in magnitude but that the overall kinetic pattern was not well described.

**Figure 8:**
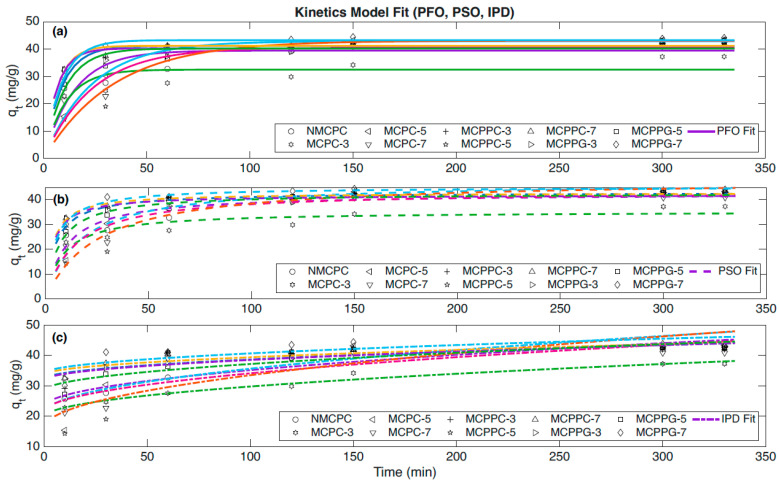
Immobilization kinetic model ((**a**) Pseudo-First-Order (PFO), (**b**) Pseudo-Second-Order (PSO) and (**c**) Intra-particular Diffusion (IPD)) of different hydrogels.

The 95% confidence intervals (95% CI) provide an estimate of the range within which the true parameter values are likely to fall with 95% certainty. Narrow confidence intervals imply greater precision in the parameter estimation, which generally enhances the reliability of the model fit. Conversely, wide intervals imply greater uncertainty and may point to variability in the experimental data or limitations of the model for that sample. In this study, samples with high R^2^ and low error values tended to exhibit narrower 95% Cis, which reinforces the conclusion that their kinetic parameters were estimated with higher confidence. Pseudo-first-order plots for all samples are shown in Figure 8. Samples with good fits displayed non-linear relationships with minimal deviation, while poorly fitting samples exhibited significant scatter.

The kinetic data for all hydrogel samples were also analyzed using the pseudo-second-order model, and the resulting calculated parameters along with statistical indicators are presented in Table 3. Model performance was evaluated using the R^2^, SSE, MSE, Chi^2^ and the 95% CI for both q_2e_ and K_2_. The pseudo-second-order rate constant (K_2_) shows the rate of immobilization toward equilibrium. Higher K_2_ values therefore exhibit faster binding kinetics, which are important for practical delivery applications. Several samples, notably MCPC-5 (R^2^ = 0.962), MCPPC-3 (R^2^ = 0.990), MCPPC-7 (R^2^ = 0.961), MCPPG-5 (R^2^ = 0.953) and MCPPG-7 (R^2^ = 0.961), demonstrated excellent model fit, indicated by very high R^2^ values (>0.94), low SSE and MSE, low Chi^2^ values and a narrow 95% CI, exhibiting high precision in parameter estimation. Narrow CI values implied that repeated experiments under similar conditions would likely yield parameter estimates very similar to the reported values, which reinforced the reliability of these fits. These results suggest that for these hydrogels, the immobilization process is possibly governed by chemisorption, involving valence forces through the sharing or exchange of electrons between BSA and the hydrogel surface. In contrast, NMCPC (R^2^ = 0.817), MCPC-3 (R^2^ = 0.712), MCPC-7 (R^2^ = 0.847), MCPPC-5 (R^2^ = 0.873) and MCPPG-3 (R^2^ = 0.814) showed poorer fits with higher SSE and MSE and wider 95% CI values. This exhibited much greater uncertainty in the estimated parameters, which implied that the pseudo-second-order model did not adequately describe the immobilization process for these hydrogel samples, possibly due to mixed or alternative kinetic mechanisms.

The intra-particle diffusion model was employed for the ten hydrogel samples to determine whether intra-particle diffusion could hold as a rate-limiting step. The kinetic data for all hydrogel samples were further examined using the IPD model with the derived parameters and related statistical measures summarized in Table 4. Model accuracy was assessed via statistical parameters R^2^, SSE, MSE, Chi^2^ and the 95% CI for both diffusion rate constant (Ki) and intercept (C). Several samples, including NMCPC (R^2^ = 0.900) MCPC-3 (R^2^ = 0.970) and MCPPG-3 (R^2^ = 0.909), exhibited excellent agreement with the model, as reflected by their high R^2^ values (>0.90), low SSE and MSE, minimal Chi^2^ values and narrow 95% CI values, indicating high precision in parameter estimation. These findings indicate that, for these hydrogels, the immobilization mechanism is likely dominated by IPD between BSA and the hydrogel surface. On the other hand, all other samples showed weaker correlation with the model, characterized by higher SSE and MSE values along with broader 95% CIs, which indicate greater uncertainty in parameter estimates. This suggests that the IPD model does not explain the immobilization kinetics for these samples.

### 3.3. Isotherm Models

The nature and process of immobilization are further explained using the Langmuir isotherm model, which assumes that substances are immobilized in a monolayer, and the Freundlich isotherm model, which characteristically describes multilayer immobilization. The Langmuir isotherm model describes the process by which liquid molecules are gradually immobilized by adsorption when they come into contact with the immobilized surface. Some of these molecules detach from the surface and allow new molecules to be immobilized swiftly. Eventually, in between this adsorption and desorption process, a dynamic equilibrium is established. Langmuir theory describes the process where immobilization occurs through a chemical adsorption process, which suggests that when all the binding sites are filled up, no more immobilization takes place.

**Figure 9:**
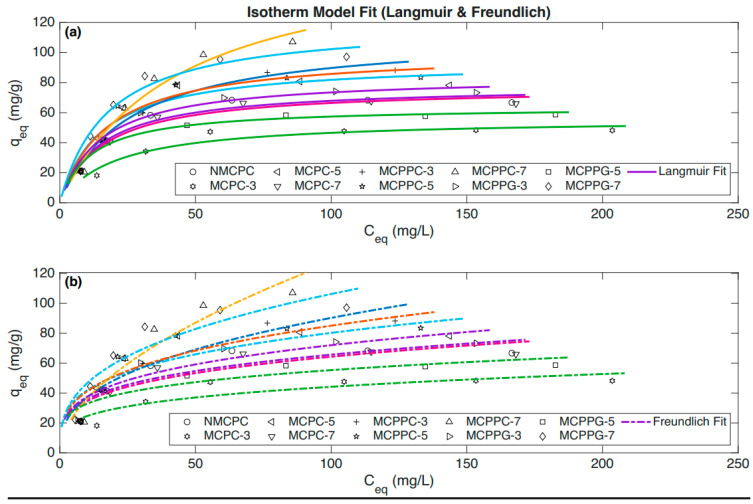
(**a**) Langmuir and (**b**) Freundlich isotherm model fitting curves of immobilization data.

Figure 9 shows the Langmuir and Freundlich isotherm model fitting curves of the immobilization data. Table 5 shows the parameters derived from fitting the experimental data of BSA immobilization onto different hydrogel samples using the Langmuir isotherm model. The immobilization data for all hydrogel samples showed excellent agreement with the Langmuir isotherm model, as evidenced by high R^2^ values exceeding 0.90. This indicates that the model effectively captures the overall adsorption behavior and trend across all samples. The Chi^2^ values ranging from 1.192 to 5.867 further reflect this variability in fit quality across samples. These relatively high error values (SSE and MSE) suggest that while the Langmuir model accurately represents the general immobilization process, there are deviations in individual measurements that are likely due to experimental variability or minor surface heterogeneities in the hydrogels. An immobilization model describes a relationship curve between the concentration of a solute on the surface of an immobilizer and the concentration of the same solute in its containing solution. $K_{f}$ and n are Freundlich constants related to maximum immobilization capacity (mg/g) and dimensionless immobilization affinity. The n value is an indicator of how favorable the immobilization process is, whereby 1 < n < 10 represents favorable adsorption. When fitting the adsorption data of the hydrogel samples to the Freundlich isotherm model, the results showed substantially weaker agreement compared to the Langmuir model.

Table 6 exhibits the parameters derived from fitting the experimental data of BSA immobilization onto different hydrogel samples using the Freundlich isotherm model. The R^2^ values were generally below 0.90, implying that the Freundlich model was less effective at capturing the overall adsorption behavior. Additionally, SSE ranged from 190.05 to 757.758 and MSE ranged from 31.675 to 126.293. These elevated error values together with comparatively high Chi^2^ values, ranging from 5.599 to 14.80, showed substantial deviations between the experimental data and the model’s predictions. This suggests that the Freundlich model does not sufficiently explain the immobilization equilibrium for these hydrogels. The weaker fit of the Freundlich model may stem from its assumptions of heterogeneous surface energies and multilayer adsorption, which appear less representative of the actual adsorption mechanism in these samples. The high error metrics imply that the model struggles to accurately reflect the adsorption intensity and capacity, which leads to greater uncertainty in the parameter estimates. For the immobilization process, the Freundlich isotherm model provides a less accurate description for these hydrogels compared to the Langmuir model, as evidenced by lower R^2^ values and substantially higher error statistics. This suggests that immobilization is more likely governed by monolayer coverage on a relatively homogeneous surface, which aligns better with the Langmuir model. In the case of the Freundlich isotherm model, the immobilization process occurs in multilayers via physical adsorption. The correlation coefficient value (R^2^) for Langmuir was found to range from 0.9781 to 0.9989, and for the Freundlich model, it ranged from 0.8108 to 0.9422. The R^2^ values further confirmed that the immobilization process was mostly fitted with the Langmuir isotherm model and adhered to pseudo-second-order kinetics. The n value within 1 to 10 suggested a favorable immobilization process as well.

### 3.4. Controlled Release of BSA

**Figure 10:**
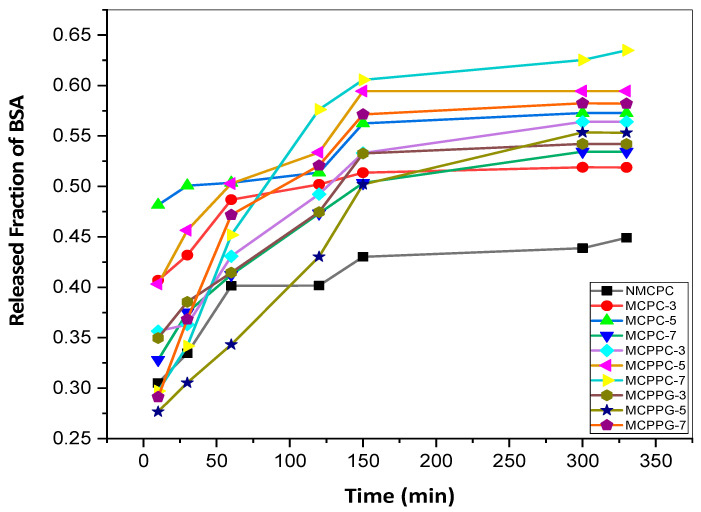
Released fraction of BSA with respect to time within a neutral pH environment.

Figure 10 shows the release of BSA from all the different hydrogels by plotting the released fraction (%) as a function of time at neutral pH. A proportional relationship between the released fraction (%) and time is evident here. With increasing time, the release of BSA also increased until it reached the saturation point at 150 min, after which there was no significant BSA release. The pore size on the hydrogel surface and hydrodynamic radii of BSA protein molecules regulate the controlled release phenomenon [29]. The equilibrium time for BSA immobilization and release is equal, which indicates that both of these phenomena are based on diffusion and electrostatic physio-chemical mutual interactivity between BSA protein molecules and glucuronic groups of the highly water-soluble hydrogel network [30].

The maximum BSA release of approximately 65% implies that a fraction of immobilized protein was retained in the hydrogel matrix. This incomplete release could be due to two primary factors. Firstly, kinetic analysis showed that numerous hydrogel formulations adhered to pseudo-second-order behavior, which suggests the predominance of chemisorption mechanisms. Robust interactions, such as hydrogen bonding or electrostatic attraction between BSA molecules and the functional groups of the CMC-based hydrogel network, could result in irreversible or partially reversible binding. Secondly, the cross-linked structure of the hydrogel may cause diffusion constraints. The pore size and network density as affected by the type and degree of crosslinking could inhibit the outward diffusion of protein molecules trapped within the internal matrix. Ultimately, the observed release profile likely shows a combination of reversible surface adsorption and partial structural entrapment in the hydrogel network.

Although there was strong agreement with the pseudo-second-order model, it should be noted that pseudo-second-order fitting by itself did not conclusively exhibit chemisorption. Kinetic models describe adsorption-rate behavior and may reflect complex or mixed interaction mechanisms. Hence, the involvement of specific chemical interactions is implied rather than definitively proven. The release behavior appears to be principally diffusion-influenced: influenced by concentration gradients and reversible electrostatic interactions between BSA and the hydrogel network. The comparable equilibrium times observed for both immobilization and release (150 min) further support a transport process regulated by molecular diffusion coupled with protein–matrix interactions rather than purely zero-order release kinetics. Given the partial release (~65%), the system likely involves an integration of diffusional transport and structural retention within the cross-linked hydrogel network.

These interpretations are consistent with the functional groups present in the CMC/PVA/PVP network and the crosslinking chemistry, which collectively govern surface charge distribution, network density and pore accessibility. Therefore, the observed immobilization–release behavior is best described as a combined physicochemical process involving electrostatic interactions, hydrogen bonding and diffusion via a physically confined hydrogel matrix.

While kinetic and isotherm analyses imply the involvement of electrostatic interactions and possible chemisorption-like behavior, these interpretations are inferred from modeling and known functional group chemistry rather than direct surface characterization. Techniques such as FTIR or XPS analysis before and after immobilization, zeta potential measurements and protein localization imaging would provide more definitive evidence and should be considered for future investigations. Similarly, this study did not evaluate hydrogel stability over multiple immobilization–release cycles, long-term structural degradation or the biological activity of BSA after release. These parameters are essential for investigating practical applicability and will be addressed in future investigations to further validate the system’s biomedical potential. Our results will be useful for researchers aiming to create drug delivery systems capable of being applied in specific medical fields, such as the development of nanocarrier systems [31].

## 4. Conclusions

This study investigated the immobilization and controlled release of BSA using microwave-synthesized EFB date palm-based hydrogels. All formulations exhibited rapid immobilization within 150 min, followed by equilibrium due to binding site saturation. Immobilization increased with initial BSA concentration prior to attaining a plateau. The process was strongly affected by electrostatic interactions relative to the isoelectric point of BSA and the hydrogel surface charge, with optimal performance observed at pH 7.4 and 37 °C. A hydrogel dosage of 2 g/L afforded the best balance between binding site availability and diffusion accessibility. Kinetic analysis indicated formulation-dependent behavior, i.e., several samples (MCPC-5, MCPPC-3, MCPPC-7, MCPPG-5, MCPPG-7) adhered to pseudo-second-order kinetics, which suggested a chemisorption mechanism, while others exhibited pseudo-first-order behavior. Equilibrium data were best described by the Langmuir model, which implied dominant monolayer adsorption on comparatively homogeneous surfaces. BSA release reached ~150 min at equilibrium and adhered to a diffusion-influenced profile governed by pore structure and protein mobility. Although promising, this study was limited to BSA as a model protein and did not include in vitro or in vivo biological evaluations. Future work should optimize CA/GA crosslinking ratios and polymer composition to better control network density and release behavior along with thorough biocompatibility and therapeutic assessments. Overall, microwave-assisted EFB-derived hydrogels demonstrate customizable adsorption and controlled release properties, which highlight their potential as sustainable biomaterials derived from EFB date palm-based waste for biomedical and pharmaceutical applications. Cytotoxicity and biocompatibility assessments were not conducted in this study and remain important areas for future investigation to establish the hydrogel’s suitability for biomedical applications.

## Figures and Tables

**Table 1:** Selection of ten samples for immobilization and controlled release.

Sample Code	Method	Chosen Sample (Blend Ratio)
NMCPC	Non-microwave (CMC/PVA/CA)	(CMC/PVA)-40/60
MCPC-3	Microwave (CMC/PVA/CA)-3 min	(CMC/PVA)-20/80
MCPC-5	Microwave (CMC/PVA/CA)-5 min	(CMC/PVA)-30/70
MCPC-7	Microwave (CMC/PVA/CA)-7 min	(CMC/PVA)-30/70
MCPPC-3	Microwave (CMC/PVA + PVP/CA)-3 min	(CMC/(PVA + PVP))-40/60
MCPPC-5	Microwave (CMC/PVA + PVP/CA)-5 min	(CMC/(PVA + PVP))-20/80
MCPPC-7	Microwave (CMC/PVA + PVP/CA)-7 min	(CMC/(PVA + PVP))-20/80
MCPPG-3	Microwave (CMC/PVA + PVP/GA)-3 min	(CMC/(PVA + PVP))-20/80
MCPPG-5	Microwave (CMC/PVA + PVP/GA)-5 min	(CMC/(PVA + PVP))-30/70
MCPPG-7	Microwave (CMC/PVA + PVP/GA)-7 min	(CMC/(PVA + PVP))-20/80

**Table 2:** Immobilization data obtained from pseudo-first-order kinetics.

Sample Name	q_1e_ * (mg/g) (95% CI)	K_1_ * (1/min) (95% CI)	R^2^	SSE	MSE	Chi^2^
NMCPC	39.43 ± 3.23 (33.10–45.76)	0.0673 ± 0.03 (0.0075–0.1271)	0.543	145.8	20.83	4.94
MCPC-3	32.48 ± 2.80 (26.99–37.98)	0.0949 ± 0.05 (0.0046–0.1943)	0.415	120.4	17.20	3.94
MCPC-5	42.98 ± 0.69 (41.61–44.34)	0.0428 ± 0.003 (0.0364–0.0495)	0.991	5.81	0.82	0.15
MCPC-7	40.67 ± 2.82 (34.92–46.42)	0.0424 ± 0.01 (0.0151–0.0697)	0.804	95.06	13.58	5.12
MCPPC-3	41.14 ± 0.82 (39.54–42.74)	0.1160 ± 0.02 (0.0859–0.1461)	0.921	10.67	1.52	0.27
MCPPC-5	43.04 ± 2.85 (37.45–48.62)	0.0296 ± 0.01 (0.0135–0.0457)	0.913	81.07	11.58	3.39
MCPPC-7	41.19 ± 1.07 (39.09–43.29)	0.1502 ± 0.03 (0.0921–0.2084)	0.772	19.19	2.74	0.47
MCPPG-3	40.21 ± 1.61 (37.06–43.36)	0.1580 ± 0.05 (0.0609–0.2550)	0.548	43.42	6.20	1.09
MCPPG-5	40.20 ± 1.74 (36.78–43.62)	0.0990 ± 0.03 (0.0461–0.1520)	0.760	47.04	6.72	1.25
MCPPG-7	43.25 ± 0.69 (41.90–44.60)	0.1168 ± 0.01 (0.0924–0.1412)	0.951	7.59	1.08	0.18

* q_1e_ and K_1_ values are presented as mean ± standard deviation (SD).

**Table 3:** Immobilization data obtained from pseudo-second-order kinetics.

Sample Name	* q_2e_ (mg/g) (95% CI)	* K_2_ (10^−3^ g/mg/min) (95% CI)	R^2^	SSE	MSE	Chi^2^
NMCPC	42.58 ± 2.73 (37.24–47.92)	2.35 ± 1.1 (0.23–4.47)	0.817	58.42	8.35	2.03
MCPC-3	35.26 ± 2.64 (30.09–40.42)	3.43 ± 2.00 (0.45–7.32)	0.712	59.18	8.45	2.11
MCPC-5	46.95 ± 2.03 (42.97–50.92)	1.29 ± 0.3 (0.62–1.96)	0.962	24.64	3.52	0.81
MCPC-7	43.61 ± 3.37 (37.01–50.22)	1.60 ± 0.8 (0.1–3.14)	0.847	74.03	10.58	2.63
MCPPC-3	42.96 ± 0.36 (42.25–43.68)	4.98 ± 0.4 (4.15–5.81)	0.990	1.35	0.19	0.03
MCPPC-5	48.15 ± 5.18 (38.00–58.31)	0.82 ± 0.5 (0.11–1.75)	0.873	117.56	16.80	3.85
MCPPC-7	42.73 ± 0.55 (41.65–43.80)	6.96 ± 1.10 (4.85–9.06)	0.961	3.28	0.47	0.08
MCPPG-3	41.89 ± 1.29 (39.36–44.41)	6.90 ± 2.5 (1.99–11.81)	0.814	17.92	2.56	0.46
MCPPG-5	42.66 ± 0.99 (40.70–44.62)	3.65 ± 0.7 (2.21–5.09)	0.953	9.30	1.33	0.26
MCPPG-7	45.11 ± 0.77 (43.60–46.62)	4.83 ± 0.8 (3.20–6.45)	0.961	6.03	0.86	0.16

* q_2e_ and K_2_ values are presented as mean ± standard deviation (SD).

**Table 4:** Immobilization data obtained from the IPD model.

Sample Name	* q_max_ (95% CI)	* K_L_ (95% CI)	R^2^	SSE	MSE	Chi^2^
NMCPC	78.13 ± 6.77 (64.86–91.40)	0.06639 ± 0.02 (0.02142–0.11137)	0.947	97.32	16.220	2.13
MCPC-3	56.12 ± 5.74 (44.86–67.37)	0.04879 ± 0.02 (0.00758–0.09000)	0.911	67.40	11.23	1.96
MCPC-5	94.34 ± 12.34 (70.17–118.2)	0.06540 ± 0.03 (0.00686–0.12395)	0.909	261.81	43.64	5.52
MCPC-7	76.96 ± 5.49 (66.21–87.72)	0.06317 ± 0.02 (0.02784–0.09850)	0.964	62.19	10.37	1.35
MCPPC-3	112.51 ± 12.68 (87.66–137.36)	0.03939 ± 0.01 (0.01433–0.06444)	0.965	127.47	21.24	2.20
MCPPC-5	100.78 ± 13.81 (73.72–127.84)	0.05717 ± 0.03 (0.00701–0.10732)	0.916	271.01	45.17	5.87
MCPPC-7	169.74 ± 33.99 (103.12–236.36)	0.02319 ± 0.009 (0.00467–0.04171)	0.962	211.50	35.25	4.41
MCPPG-3	84.59 ± 5.14 (74.52–94.67)	0.06591 ± 0.02 (0.03563–0.09620)	0.976	52.04	8.67	1.19
MCPPG-5	64.00 ± 3.98 (56.21–71.80)	0.08687 ± 0.03 (0.03657–0.13717)	0.954	49.75	8.29	1.51
MCPPG-7	120.24 ± 12.29 (96.15–144.33)	0.05684 ± 0.02 (0.02295–0.09074)	0.966	153.82	25.64	2.96

* q_max_ and K_L_ values are presented as mean ± standard deviation (SD).

**Table 5:** Langmuir model constants for BSA immobilization.

Sample Name	* q_max_ (95% CI)	* K_L_ (95% CI)	R^2^	SSE	MSE	Chi^2^
NMCPC	78.13 ± 6.77 (64.86–91.40)	0.06639 ± 0.02 (0.02142–0.11137)	0.947	97.32	16.220	2.13
MCPC-3	56.12 ± 5.74 (44.86–67.37)	0.04879 ± 0.02 (0.00758–0.09000)	0.911	67.40	11.23	1.96
MCPC-5	94.34 ± 12.34 (70.17–118.2)	0.06540 ± 0.03 (0.00686–0.12395)	0.909	261.81	43.64	5.52
MCPC-7	76.96 ± 5.49 (66.21–87.72)	0.06317 ± 0.02 (0.02784–0.09850)	0.964	62.19	10.37	1.35
MCPPC-3	112.51 ± 12.68 (87.66–137.36)	0.03939 ± 0.01 (0.01433–0.06444)	0.965	127.47	21.24	2.20
MCPPC-5	100.78 ± 13.81 (73.72–127.84)	0.05717 ± 0.03 (0.00701–0.10732)	0.916	271.01	45.17	5.87
MCPPC-7	169.74 ± 33.99 (103.12–236.36)	0.02319 ± 0.009 (0.00467–0.04171)	0.962	211.50	35.25	4.41
MCPPG-3	84.59 ± 5.14 (74.52–94.67)	0.06591 ± 0.02 (0.03563–0.09620)	0.976	52.04	8.67	1.19
MCPPG-5	64.00 ± 3.98 (56.21–71.80)	0.08687 ± 0.03 (0.03657–0.13717)	0.954	49.75	8.29	1.51
MCPPG-7	120.24 ± 12.29 (96.15–144.33)	0.05684 ± 0.02 (0.02295–0.09074)	0.966	153.82	25.64	2.96

* q_max_ and K_L_ values are presented as mean ± standard deviation (SD).

**Table 6:** Freundlich model constants for BSA immobilization.

Sample Name	* K_F_ (95% CI)	* n (95% CI)	R^2^	SSE	MSE	Chi^2^
NMCPC	19.60 ± 9.73 (0.542–38.67)	3.808 ± 1.65 (0.565–7.050)	0.780	401.94	66.99	8.7
MCPC-3	13.68 ± 7.62 (1.26–28.63)	3.929 ± 1.85 (0.294–7.564)	0.748	190.05	31.68	5.60
MCPC-5	21.71 ± 12.23 (2.26–45.68)	3.521 ± 1.68 (0.232–6.811)	0.738	757.758	126.29	14.80
MCPC-7	18.55 ± 8.63 (1.63–35.47)	3.704 ± 1.46 (0.848–6.560)	0.814	322.30	53.72	7.20
MCPPC-3	15.29 ± 7.86 (0.12–30.70)	2.594 ± 0.83 (0.965–4.222)	0.870	474.40	79.07	8.88
MCPPC-5	19.92 ± 11.19 (2.01–41.85)	3.172 ± 1.36 (0.497–5.846)	0.776	726.364	121.06	14.46
MCPPC-7	10.25 ± 5.86 (1.24–21.73)	1.828 ± 0.49 (0.875–2.781)	0.905	535.595	89.27	9.86
MCPPG-3	19.46 ± 8.38 (3.04–35.87)	3.518 ± 1.24 (1.087–5.950)	0.845	339.921	56.65	7.38
MCPPG-5	19.45 ± 7.69 (4.39–34.53)	4.406 ± 1.72 (1.037–7.775)	0.814	202.247	33.71	5.34
MCPPG-7	20.35 ± 9.80 (1.14–39.56)	2.786 ± 0.95 (0.925–4.647)	0.850	681.390	113.57	12.10

* K_F_ and n values are presented as mean ± standard deviation (SD).

## Data Availability

The original contributions presented in this study are included in the article. Further inquiries can be directed to the corresponding author.
